# Glaucoma patients have an increased level of trimethylamine, a toxic product of gut bacteria, in the aqueous humor: a pilot study

**DOI:** 10.1007/s10792-020-01587-y

**Published:** 2020-09-11

**Authors:** Janusz Skrzypecki, J. Izdebska, A. Kamińska, J. Badowska, J. Przybek-Skrzypecka, J. Bombuy, E. Samborowska, J. P. Szaflik

**Affiliations:** 1 Laboratory of Centre for Preclinical Research, Department of Experimental Physiology and Pathophysiology, Medical University of Warsaw, Banacha 1B, 02-097 Warsaw, Poland; 2 Department of Ophthalmology, SPKSO Ophthalmic Hospital, Medical University of Warsaw, Warsaw, Poland; 3 Mass Spectrometry Laboratory, Institute of Biochemistry and Biophysics, Polish Academy of Sciences, Warsaw, Poland

**Keywords:** TMA, TMAO, Intraocular pressure, Betaine

## Abstract

**Purpose:**

Animal studies suggest that gut bacteria metabolites are involved in regulation of intraocular pressure or development of glaucoma. However, clinical data are lacking. Here, we wanted to compare level of trimethylamine (TMA), an uremic toxin produced by gut bacteria, along with betaine and trimethylamine N-oxide (TMAO), a substrate and a product of its metabolism, in the aqueous humor and in plasma of patients with glaucoma and their controls.

**Methods:**

Twenty patients were selected for cataract phacoemulsification, and 20 patients selected for phacotrabeculectomy were enrolled in the study. Patients were matched with controls on systemic diseases and estimated glomerular filtration rate. Blood samples were collected in the preoperative suite, whereas aqueous humor samples were collected as the first step of both procedures. Subsequently, level of betaine, TMA and TMAO was analyzed by means of chromatography.

**Results:**

In the aqueous humor, level of TMA, but not betaine or TMAO, was significantly higher in the phacotrabeculectomy group than in the phacoemulsification group. Plasma level of betaine, TMA and TMAO was similar between groups. In both groups, level of betaine and TMA, but not TMAO, was significantly higher in plasma than in the aqueous humor.

**Conclusion:**

TMA, but not TMAO or betaine level, is increased in the aqueous humor of patients with glaucoma. TMA might play a role in pathogenesis of glaucoma; however, prospective studies are needed to confirm our findings.

## Introduction

The most recent studies prove that bacterial metabolites are involved in pathophysiology of many diseases [1]. Their role in multiple sclerosis, hypertension, heart failure or cancer has been highlighted [2]. Furthermore, recent evidence implies that gut bacterial metabolites, including short-chain fatty acids (SCFA), hydrogen sulfide and trimethylamine N-oxide (TMAO), play an important role in regulation of intraocular pressure (IOP) [3–5].

In vitro experiments suggest that TMAO, a product of betaine, choline and carnitine metabolism, stabilizes mutant myocilin and prevents degradation of trabecular meshwork in the juvenile, hereditary glaucoma [4]. Observations regarding TMAO were linked to its chaperonic activity [4]. Notably, TMAO protects cardiomyocytes from increased load in the setting of hypertension or heart failure in rats and acts as a buffer against increased osmotic and hydrostatic pressure in the deep-sea animals [6–8]. In contrast, trimethylamine (TMA), a substrate for TMAO synthesis, exerts cytotoxic effect on vascular smooth muscles and cardiomyocytes [9, 10].

Interestingly, role of TMA and TMAO in primary open-angle glaucoma (POAG) has not been thoroughly investigated in clinical studies so far. It is possible that TMAO acting as a chaperone might protect proteins in the outflow pathways as well as in the optic nerve fibers from pressure-related degeneration [11]. Contrarily, TMA might negatively affect optic fibers and smooth muscle cells in the trabecular meshwork [12]. These negative effects of TMA might be primarily related to its effects on lactate dehydrogenase, an enzyme involved in protection against hypoxic stress—one of causative factors for development and progression of glaucoma [12–14].

Furthermore, although various cardiovascular diseases, ischemic heart disease or hypertension, are linked to development of glaucoma, mechanistic relationship between these seemingly distinct entities is far from clear. We believe that TMA and TMAO might constitute a common humoral background which affects not only cardiovascular system, but also has a direct or indirect effect on the structure of the optic nerve. Firstly, TMA and TMAO are involved in blood pressure (BP) homeostasis [15–17]. Notably, dysregulation of BP is considered a risk factor for both cardiovascular mortality and glaucoma [18, 19]. Secondly, TMAO was shown to accelerate development of atherosclerosis which is considered a risk factor for glaucoma [20–22].

Here, we evaluated the levels of TMA, TMAO and betaine, a TMA precursor, in the aqueous humor and in the blood of patients with increased and normal IOP.

## Materials and methods

This cross-sectional study was approved by the Bioethical Committee at the Medical University of Warsaw and adhered to the Declaration of Helsinki. All patients were enrolled at the Department of Ophthalmology at the Medical University of Warsaw, a tertiary eye care center in Poland.

Considering the preliminary character of the study and lack of sufficient data to calculate power of the study number of participants was arbitrarily set at 20 in both groups.

### Inclusion criteria

The first group consisted of patients undergoing routine cataract surgery. Patients with advanced primary open-angle glaucoma, who were selected for combined procedure of phacoemulsification and trabeculectomy, were included in the second group. Considering that level of TMA was shown to be inversely proportional to estimated glomerular filtration rate (eGFR) [10], whereas level of TMAO correlates with cardiovascular mortality; we decided to match patients on eGFR as well as diseases related to cardiovascular mortality, i.e., diabetes, hypertension and ischemic heart disease. To calculate eGFR, we utilized Modification of Diet in Renal Disease (MDRD) study equation.

Both groups underwent routine slit-lamp examination, including IOP measurement and optic nerve disc assessment. Patients were selected for phacotrabeculectomy procedure based on increased cup-to-disc ratio, elevated IOP and other morphological or functional features of glaucomatous neuropathy, including visual field or optical coherence tomography examination.

### Exclusion criteria

Conditions which might impair blood–aqueous humor barrier were set as exclusion criteria, including uveitic or traumatic cataract and proliferative diabetic retinopathy. Furthermore, patients with known gastrointestinal disorders which increase permeability of gut–blood barrier and patients who underwent treatment with antibiotics 3 months prior to the study were excluded as well. Additionally, patients treated for glaucoma were not included in the control group.

### Collection of aqueous humor sample

Aqueous humor was collected at the beginning of the procedure. Following a clear corneal, 1 mm paracentesis 30G cannula was introduced to the anterior chamber of the eye and 100 μl of the aqueous humor was aspirated. In order to avoid contamination of the aqueous humor with blood, care was taken not to injure limbal vessels. Following collection of fluid, both phacoemulsification and phacotrabeculectomy procedures were performed as usual. Aqueous humor was stored at – 20 °C before analysis.

### Collection of peripheral blood sample

Sample of venous blood was collected to a vial containing EDTA. Collection was performed in the preoperative suite before infusion of sedating drugs; 2 ml of blood was centrifuged at 5000/min for 10 min, and serum was aspirated and transferred to an empty vial which was subsequently stored at – 20 °C before analysis.

### Betaine, TMA and TMAO concentration evaluation (chromatography)

Chemicals The following chemicals were used: LC–MS grade—acetonitrile, HPLC gradient grade acetone (POCh), 25% ammonium hydroxide and formic acid (J.T. Baker). Ultra-pure water was obtained from water purification system (Milli-Q, Millipore). TMA, TMAO and betaine hydrochloride were purchased from Sigma-Aldrich. All stock solutions were prepared in methanol.

Sample preparation It was performed as follows: 10 μl of sample was mixed with 100 μl of acetone containing internal standards. After the mixture was vortexed and centrifuged. A 7 μl of aliquot was injected into apparatus.

Analytes They were quantified at the Institute of Biochemistry and Biophysics using liquid chromatography–tandem mass spectrometry method. The instrumentation consisted of a Waters Acquity Ultra-Performance Liquid Chromatograph coupled with Waters TQ-S triple quadrupole mass spectrometer. Chromatographic separation was performed using a Waters HILIC column (1.7 µm, 2.1 mm × 50 mm) thermostated at 70 °C. Mobile phase A was Milli-Q water with addition of 1 ml of 25% NH4OH per 1000 ml of water, and mobile phase B was 1 ml of formic acid in 1000 ml of acetonitrile. The flow rate of mobile phase was set at 0.5 ml/min. The total time of separation was 1.7 min. The mass spectrometer operated in multiple-reaction monitoring (MRM) negative electrospray ionization (ESI−) mode for indoxyl sulfate and in multiple-reaction monitoring (MRM) positive electrospray ionization (ESI+) mode for other analytes. The calibration curve ranges were 0.02–20 µg/ml for TMAO, 0.1–120 µg/ml for TMA, 0.1–50 µg/ml for indoxyl sulfate and 0.02–10 µg/ml. Mean R2 coefficients of a calibration curves from six calibrators were not lower than 0.99.

### Statistical analysis

Data were analyzed for normal distribution with Kolmogorov–Smirnov test. Differences between groups were tested for statistical significance with unpaired Student *t* test. Contingency tables were analyzed with Cochran’s Q test. Level of statistical significance was set at 0.05. All analyses were performed with Graphpad (Prism, USA) and Excel (Microsoft, USA).

## Results

Characteristics of the studied population are included in Table 1. There was a statistically significant difference in IOP between phacoemulsification and phacotrabeculectomy groups (*P* < 0.001). There were no statistically significant differences in age, eGFR, number of patients with diabetes, hypertension or ischemic heart disease between groups.

**Table 1:** Characteristics of the group

	Phacoemulsification	Phacotrabeculectomy	*P*
Number	20	20	*P* > 0.05
Age	73.7 ± 1.8	72.1 ± 1.9	*P* > 0.05
IOP (mmHg)	13.3 ± 0.8	21.5 ± 1.6	*P* < 0.05
Creatinine (mg/ml)	0.82 ± 0.04	0.84 ± 0.04	*P* < 0.05
eGFR (ml/min/1.73 m^2^)	87 ± 6	90 ± 5	*P* > 0.05
Cup-to-disc ratio	0.3 ± 0.1	0.8 ± 0.2	*P* < 0.05
Number of anti-glaucoma drugs	0	3 ± 0.2	*P* < 0.05
Mean deviation (visual field)	0.2 ± 0.5	6.2 ± 1.2	*P* < 0.05
LOCS III nuclear score	3 ± 0.3	3.2 ± 0.35	*P* > 0.05
*Diseases related to cardiovascular mortality*			
Hypertension	16	14	*P* > 0.05
Ischemic heart disease	5	3	*P* > 0.05
Diabetes	4	5	*P* > 0.05
*Classes of anti-glaucoma medication*			
Prostaglandins	0	16	*P* < 0.05
Beta blockers	0	14	*P* < 0.05
Alpha-2 agonists	0	15	*P* < 0.05
Anhydrase inhibitors	0	16	*P* < 0.05

Results are expressed as an absolute number or a mean ± standard error of the mean (SEM)

LOCS III—The lens opacities classification system

### Plasma and aqueous humor levels of TMA, betaine and TMAO

**Fig. 1:**
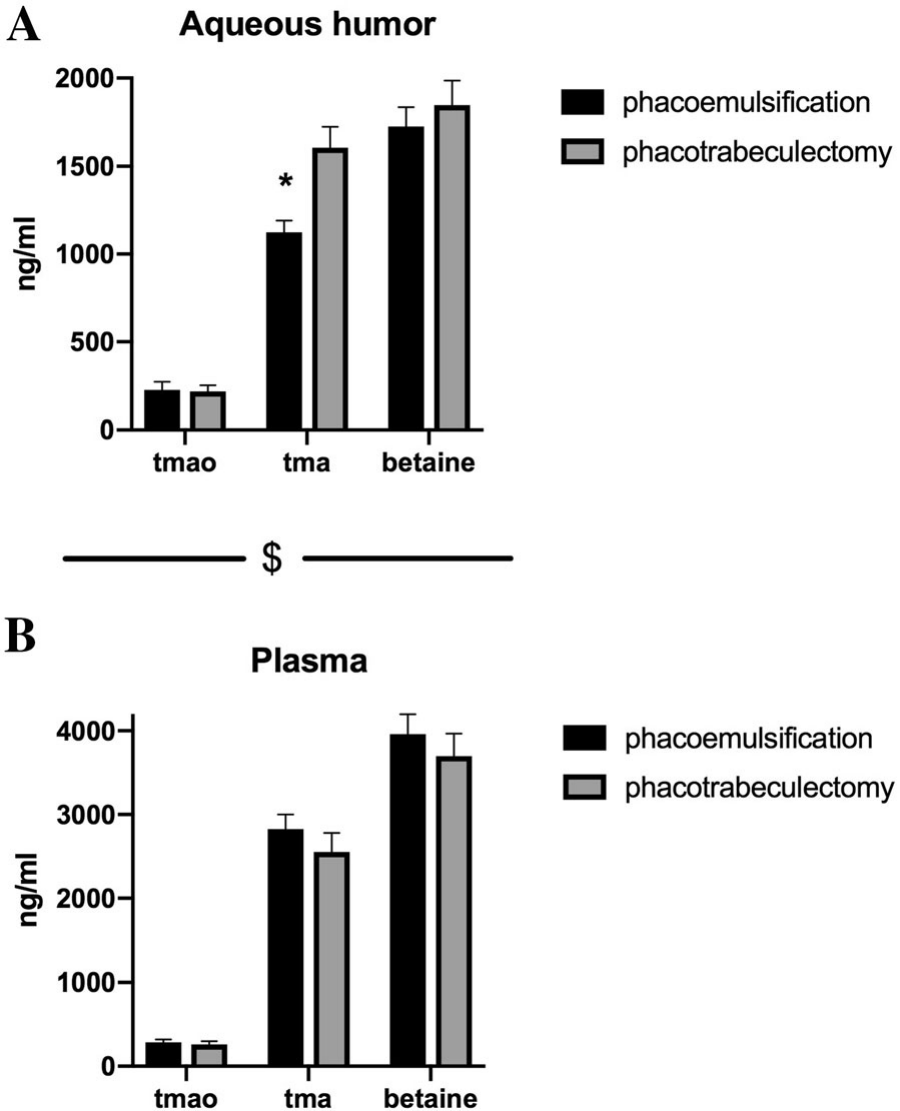
**a** Aqueous humor level of trimethylamine (TMA), trimethylamine N-oxide (TMAO) and betaine. **b** Plasma level of trimethylamine (TMA), trimethylamine N-oxide (TMAO) and betaine. *p < 0.05 phacoemulsification TMA versus phacotrabeculectomy TMA. ^$^*p* < 0.05 plasma TMA versus aqueous humor TMA; plasma betaine versus aqueous humor betaine

Plasma level of TMA and betaine was significantly higher than aqueous humor level of TMA and betaine in both groups (phacotrabeculectomy betaine *P* < 0.001; TMA *P* = 0.001) (phacoemulsification betaine *P* < 0.001; TMA *P* < 0.001). There was no difference between aqueous humor and plasma level of TMAO (Fig. 1a, b).

### Phacoemulsification versus phacotrabeculectomy

Aqueous humor level of TMA, but not betaine or TMAO, was significantly higher (*P* < 0.001) in phacotrabeculectomy group than in phacoemulsification group (Fig. 1a). There were no significant differences between plasma level of TMA, betaine and TMAO in phacotrabeculectomy and phacoemulsification groups (Fig. 1b).

## Discussion

Here, we have found that the aqueous humor contains TMA, a gut bacteria metabolite, as well as a substrate and a product of its metabolic pathway, i.e., betaine and TMAO, respectively. Furthermore, we have shown that level of TMA in the aqueous humor of patients with advanced open-angle glaucoma is significantly higher than in phacoemulsification control group.

Betaine, l-carnitine and choline are abundant in red meat and in deep-sea animals [23, 24]. They are metabolized by gut bacteria to TMA, which is in turn oxygenated to TMAO in the liver [25]. Additionally, saltwater fish constitute an important direct dietary source of TMAO [26].

The landmark study which linked an increased level of TMAO to cardiovascular mortality triggered interest in hypothesis that products of l-carnitine and betaine metabolism, which involves gut bacteria, play a pivotal role in maintenance of homeostasis [27, 28]. Basic and clinical research which followed proved that TMAO prolongs effects of angiotensin II and plays a role in carcinogenesis or development of diabetes [26].

However, growing body of research shows that it is not TMAO, which is a product of liver oxygenation of TMA, but rather TMA itself is toxic and might be responsible for decreased viability of smooth muscles or cardiomyocytes and may directly lead to dysregulation of homeostasis [10].

To the best of our knowledge, no previous clinical study investigated the presence of gut bacteria metabolites in the aqueous humor of patients with cataract and patients with cataract and glaucoma.

Here, we have found that betaine, TMA and TMAO are present in the aqueous humor of cataract patients and patients with both cataract and glaucoma. Interestingly, although level of TMAO in the aqueous humor and in the plasma is similar, concentration of TMA and betaine in the eye is twofold lower than in the plasma. Considering that the exclusive locations of TMAO and TMA synthesis are the liver and the gut, respectively [29], our findings suggest that TMAO diffuses freely from blood to the aqueous humor and does not undergo metabolism in the eye. In contrast, distribution of TMA and betaine to the eye might be restricted by specific transporters, active removal or metabolism.

Furthermore, we have found that level of TMA in the aqueous humor of glaucoma patients is significantly higher than in cataract patients. Considering that TMA has been shown to impair smooth muscle function [10], we hypothesize that TMA might negatively affect smooth muscle cells in the trabecular meshwork and decrease filtration. What is more, TMA was found to accelerate degeneration of lactate dehydrogenase (LDH) [10]. It is well established that mechanisms involved in response to hypoxia, including LDH, are impaired in glaucomatous eyes [13, 14]. We hypothesize that TMA might further interfere with defense mechanisms against hypoxic stress and accelerate development or progression of glaucoma.

Furthermore, large cross-sectional studies showed positive correlation between chronic kidney disease and incidence of open-angle glaucoma [30, 31]. Notably, given that plasma level of TMA is inversely correlated with eGFR, TMA might constitute a plausible mechanistic link between chronic kidney disease and glaucoma [10].

Limitations of our study should be underlined. Firstly, considering that patients selected for phacotrabeculectomy procedure were under influence of anti-glaucoma medication, we cannot rule out effects of these drugs on level of betaine, TMA and TMAO, particularly in the aqueous humor. Secondly, we cannot rule out influence of other factors, including diet or baseline IOP in the phacotrabeculectomy group. Finally, prospective and randomized trials are needed to fully elucidate role of gut bacteria metabolites in the development and progression of glaucoma.

In conclusion, TMA, but not TMAO or betaine level is increased in the aqueous humor of patients with glaucoma. TMA might play a role in pathogenesis of glaucoma, however, prospective studies are needed to confirm our findings.

## Data Availability

All relevant data are included in the manuscript.
